# Belimumab Decreases Autophagy and Citrullination in Peripheral Blood Mononuclear Cells from Patients with Systemic Lupus Erythematosus

**DOI:** 10.3390/cells11020262

**Published:** 2022-01-13

**Authors:** Tania Colasanti, Francesca Romana Spinelli, Cristiana Barbati, Fulvia Ceccarelli, Susanna Scarpa, Marta Vomero, Cristiano Alessandri, Guido Valesini, Fabrizio Conti

**Affiliations:** 1 Rheumatology Unit, Department of Clinical Internal, Anesthesiological and Cardiovascular Sciences, Sapienza University of Rome, Viale del Policlinico, 155, 00161 Rome, Italy; francescaromana.spinelli@uniroma1.it (F.R.S.); cristiana.barbati1@gmail.com (C.B.); fulvia.ceccarelli@uniroma1.it (F.C.); marta.vomero@gmail.com (M.V.); cristiano.alessandri@uniroma1.it (C.A.); guido.valesini@uniroma1.it (G.V.); fabrizio.conti@uniroma1.it (F.C.); 2 Department of Experimental Medicine, Sapienza University of Rome, Viale del Policlinico, 155, 00161 Rome, Italy; susanna.scarpa@uniroma1.it

**Keywords:** belimumab, autophagy, citrullination, peptidylarginine deiminases (PADs), systemic lupus erythematosus, IL-18, B lymphocyte stimulator (BLyS), BLyS receptors

## Abstract

Belimumab (BLM) is a B lymphocyte stimulator (BLyS) inhibitor approved for the treatment of systemic lupus erythematosus (SLE). Autophagy is a cell survival mechanism involved in the pathogenesis of SLE. Citrullination is a post-translational modification catalyzed by peptidylarginine deiminase (PAD) enzymes. Autophagy and citrullination may generate neoepitopes, evoking an autoimmune response. No previous studies have investigated the connection of these processes, and how BLM could affect them, in SLE. Ex vivo autophagy and protein citrullination were analyzed by western blot in lysates from 26 SLE patients’ PBMCs at baseline and after 2, 4, and 12 weeks of BLM administration, and from 16 healthy donors’ PBMCs. Autophagic PBMCs were identified by the immunofluorescent detection of the autophagy-associated proteins LC3B (LC3 puncta) and LAMP-1. Autophagosome accumulation was evaluated in CD14^−^ (PBLs) and CD14^+^ (monocytes) SLE cells. The presence of the BLyS receptors BAFF-R, BCMA, and TACI on SLE CD4^+^, CD8^+^ T cells and monocytes, as well as serum IL-18 levels, was also assessed. Following BLM administration, we observed a decrease in autophagy and citrullination, with a lowering of LC3-II, citrullinated vimentin, and PAD4 expression levels in PBMCs from SLE patients. LC3-II levels showed a correlation with the SLE Disease Activity Index 2000 (SLEDAI-2K) after 12 weeks of therapy. The LC3B/LAMP-1 analysis confirmed the reduction in autophagy. A lesser autophagosome accumulation occurred in PBLs and monocytes which, in turn, seemed to be the main cellular populations contributing to autophagy. A reduction in patients’ serum IL-18 concentrations occurred. CD4^+^ and CD8^+^ cells weakly expressed BAFF receptors; monocytes expressed only BAFF-R. BLM could impact on autophagy and citrullination, offering an opportunity for a deeper understanding of these mechanisms in SLE, and a possible tool for the clinical management of SLE.

## 1. Introduction

Systemic lupus erythematosus (SLE) is a chronic autoimmune disease caused by a dysregulation of innate and adaptive immune cells, ultimately resulting in tissue and organ damage. Many findings from animal models and genetic studies show that autophagy is involved in the onset, progression, and severity of SLE [1]. Genome-wide association studies found that single-nucleotide polymorphisms in autophagy-related genes (*Atgs*)—in particular *Atg5*—are linked to susceptibility to SLE. Indeed, the impairment of *Atg5*-dependent mechanisms—especially the clearance of dying cells and cell antigen presentation—might be a concurrent cause of autoimmunity and inflammation [2].

Autophagy is a highly preserved degradation process, whereby discarded cytoplasm contents are enveloped by the double-membrane autophagosomes and degraded after delivery to the lysosomes. In this way, the process of autophagy recycles nutrients and cellular components, thus maintaining cellular homeostasis, as well as cell survival during stress conditions, in which it can be impaired [1]. Autophagy can be involved in inflammatory processes, affecting the activation of inflammasomes and the secretion of inflammatory mediators [3,4].

Epigenetic mechanisms are considered to be key players in many biological processes and disease conditions, such as tumor development and progression [5,6], and citrullination (as a covalent histone post-translational modification (PTM)) has been extensively investigated in epigenetic studies [7]. PTMs such as citrullination, carbamylation, and homocysteinylation can result in conformational changes driving inflammation and autoimmune responses [8,9]. Protein citrullination is catalyzed by peptidylarginine deiminases (PADs)—tissue-specific enzymes involved in the conversion of arginine to citrulline. Citrullinated proteins can be recognized as “non-self”, triggering an autoimmune response, and antibodies targeting citrullinated proteins have been detected in autoimmune diseases, such as rheumatoid arthritis (RA) and SLE [10,11]. Particularly, anti-carbamylated protein (anti-CarP) antibodies and anti-citrullinated protein antibodies (ACPA) have been shown to be able to represent biomarkers of severity (erosivity) and to trigger and maintain the inflammation status in SLE [12,13]. In this context, autophagy is involved in the generation of citrullinated peptides by antigen-presenting cells [14,15]. Moreover, citrullination is the protein end-of-life and degradation “tag”, consequently connected with cell survival mechanisms and, hence, with autophagy. From the chemical point of view, PAD-mediated citrullination increases the hydrophobicity of the proteins, converting arginine residues (positively charged at a neutral pH) into citrulline, which has no net charge. This can lead to protein unfolding and loss of functionality, and autophagy is the assigned mechanism for unfolded protein degradation.

B lymphocyte stimulator (BLyS) is a crucial cytokine in the pathogenesis of SLE, contributing to B cells’ survival and consequent autoantibody production [16]; in addition, BLyS levels are correlated with SLE disease activity. Aside from B cells, T cells are also susceptible to the effects of BLyS [17], expressing the following receptors for BLyS: BLyS receptor 3 (BR3, also known as B-cell activating factor receptor (BAFF-R)), B-Cell maturation antigen (BCMA), and transmembrane activator-1 and calcium modulator and cyclophilin ligand interactor (TACI).

The binding of BLyS to BAFF-R results in activation of the non-canonical nuclear factor kappa-light-chain-enhancer of activated B cells (NF-κB) signaling pathway, leading to maturation and survival of B cells. This signaling pathway also involves the activation of inhibitor of NF-κB kinase (IKK) and c-Jun *N*-terminal kinase 1 (JNK1), so that it can activate autophagy pathways. BAFF signaling through TACI and BCMA can also activate IKK, potentially modulating downstream autophagy, leading to plasma cell maintenance and survival [18,19].

To date, there is no evidence on the effects of SLE therapy on either autophagy or citrullination—an autophagy-related PTM. Hence, the aim of this study was to evaluate changes in autophagy and protein citrullination pathways ex vivo in peripheral blood mononuclear cells (PBMCs) from SLE patients undergoing treatment with belimumab (BLM)—a BLyS-specific inhibitor. Autophagic PBMCs were also identified by the colocalization of the autophagosome protein microtubule-associated protein 1A/1B-light chain 3 (LC3B), measured by immunofluorescence (IF) as a punctate pattern named LC3 puncta stain, and the lysosomal-associated membrane protein-1 (LAMP-1). The presence of BLyS receptors on CD4^+^, CD8^+^ T lymphocytes and CD14^+^ cells (monocytes) from SLE patients was also assessed. Finally, since autophagy seems to be involved in facilitating the release of IL-1 family cytokines, and recent studies have investigated the role of IL-18 as a relevant pro-inflammatory cytokine in SLE [20], IL-18 levels were also evaluated in patients’ sera.

## 2. Materials and Methods

### 2.1. Selection of Patients and Samples

We enrolled consecutive patients fulfilling the American College of Rheumatology (ACR) 1997 revised criteria for SLE [21] candidates for BLM, because of unresponsiveness to standard therapy. As required by the Italian regulations, all patients starting BLM had an active disease and a serum positivity for anti-double-strand (ds) DNA antibodies (Abs), either by Farr assay, indirect immunofluorescence (IF), or enzyme-linked immunosorbent assay (ELISA), and/or low complement (C)3 or C4 levels.

Our study also included 16 healthy donors (HDs) not diagnosed with any autoimmune and/or inflammatory disorders.

All patients underwent venous blood draw at the baseline, and after 2, 4, and 12 weeks of therapy with BLM. PBMCs were isolated on the same day via density gradient centrifugation (Lympholyte-H; Cedarlane Laboratories, Burlington, Ontario, Canada).

After collection, the whole blood was left at room temperature for 15–30 min and allowed to clot. Then, the clots were removed by centrifuging at 2000× *g* for 10 min in a refrigerated centrifuge (5804 R; Eppendorf, Hamburg, Germany). The serum samples obtained were aseptically aliquoted and stored at −80 °C until the assay.

Demographic and clinical data were recorded at the baseline visit; moreover, at each visit (baseline, and after 2, 4, and 12 weeks), SLE disease activity was evaluated by the SLE Disease Activity Index 2000 (SLEDAI-2K).

The procedures involving human participants were in accordance with the Declaration of Helsinki. The study protocol was approved by the Bioethics Committee of the Sapienza University of Rome (Prot. 120/16). An informed consent was obtained from all individual participants included in the study.

### 2.2. Sodium Dodecyl Sulphate–Polyacrylamide Gel Electrophoresis (SDS–PAGE) and Immunoblotting

In order to evaluate autophagy and protein citrullination levels, PBMCs were lysed in lysis buffer (100 mM Tris-HCl, pH 8; 150 mM NaCl; 1% Triton X-100; 1 mM MgCl_2_; 25 mM Na_3_VO_4_), in the presence of Complete Protease Inhibitor Mixture (Roche Diagnostics, Deutschland GmbH, Mannheim, Germany). Protein content was determined by the Bradford assay (Bio-Rad Laboratories, Hercules, CA, USA). Lysates (60 µg) were run onto a 4–15% gradient SDS–PAGE (Bio-Rad Laboratories, Hercules, CA, USA) in denaturing conditions and, after electrophoresis, proteins were transferred onto polyvinylidene difluoride membrane (PVDF; Amersham Hybond-ECL, GE Healthcare Italy, Milan, Italy) by means of a Trans-Blot transfer cell (Bio-Rad Laboratories, Hercules, CA, USA). If needed, the membranes were cropped at the molecular weight of interest, in order to enhance the antibody yield. For detection of citrullinated proteins and the marker of lymphocyte autophagy α-synuclein (hereafter referred to as α-syn), and in order to improve protein retention (because these proteins tend to easily detach from blotted membranes), the method described by Colasanti et al. was used [22]. The membranes were then blocked in 5% nonfat dried milk (Euroclone S.p.A., Pero, Milan, Italy) in Tris-buffered saline containing 0.1% Tween 20 (TBS-T) for 1 h at room temperature, and then rinsed, and incubated with the intended Abs. As primary Abs, rabbit IgG anti-LC3B (hereafter referred to as LC3-II; LC3B D11 XP Rabbit mAb, Cell Signaling Technology, Danvers, MA, USA), anti-p62/SQSTM (hereafter referred to as p62; Sigma-Aldrich, St. Louis, MO, USA), and anti-PADI4/PAD4 (hereafter referred to as PAD4; Abcam, Cambridge, UK) Abs were used at a dilution of 1:1000 in TBS-T containing 5% bovine serum albumin (BSA; Sigma-Aldrich, St. Louis, MO, USA). F95 mouse IgM anti-citrullinated proteins (Anti-peptidyl-citrulline, clone F95, Merck Millipore, Darmstadt, Germany) and mouse monoclonal anti-α-syn (clone Syn211, Sigma-Aldrich, St. Louis, MO, USA) were used at a dilution of 1:500 in TBS-T with 5% nonfat dried milk (Euroclone S.p.A., Pero, Milan, Italy).

Excess primary antibodies were removed by washing the PVDF membranes in TBS-T. The membranes were then incubated with horseradish peroxidase (HRP)-conjugated goat anti-rabbit IgG (Bio-Rad Laboratories, Hercules, CA, USA) diluted 1:3000, or with goat anti-mouse IgM (Jackson ImmunoResearch Europe Ltd., Ely, Cambridgeshire, UK) diluted 1:5000, both in TBS-T with 5% nonfat dried milk (Euroclone S.p.A., Pero, Milan, Italy). The reactions were developed using the chemiluminescent HRP detection reagent Luminata Forte (Merck Millipore, Darmstadt, Germany). A rabbit IgG anti-β-actin Ab (Sigma-Aldrich, St. Louis, MO, USA) was used for protein content control.

Immunoprecipitated samples (30 μg) were equally subjected to 4–15% gradient SDS–PAGE in denaturing conditions and immunoblotting, using rabbit IgG anti-vimentin as a control (Vimentin D21H3 XP Rabbit mAb, Cell Signaling Technology, Danvers, MA, USA; dilution 1:1000 in TBS-T containing 5% BSA) and F95 mouse IgM anti-citrullinated proteins (used as previously indicated).

Quantification of protein expression was performed by densitometric analysis of the autoradiograms (GS-700 Imaging Densitometer, Bio-Rad Laboratories, Hercules, CA, USA). The results shown are expressed as the mean  ±  standard deviation (SD).

### 2.3. Immunoprecipitation

Lysates from SLE (at baseline, and after 2, 4, and 12 weeks of treatment with BLM) and HD PBMCs were used for immunoprecipitation, using rabbit IgG anti-vimentin or irrelevant IgG (Irr. IgG; 10 μg each) as controls. In brief, following manufacturer’s instructions for the Thermo Fisher Scientific Pierce Direct IP Kit (Pierce Biotechnology, Waltham, MA, USA), PBMCs were lysed in lysis buffer as previously described and, in order to preclear nonspecific binding, cell-free lysates were mixed with control agarose resin slurry and stirred in a rotary shaker for 1 h at 4 °C. After centrifugation (1000× *g* for 1 min), vimentin was immunoprecipitated from the precleared samples.

### 2.4. Immunofluorescence (IF)

Autophagy was evaluated through the detection of the autophagosome marker LC3B and colocalization with the lysosome marker LAMP-1. A blinded image analysis was performed by an expert pathologist.

An indirect IF assay was performed on PBMCs from SLE patients at baseline, and after 2, 4, and 12 weeks of treatment with BLM, and from HDs, immobilized on slides by cytospin centrifugation. All dilutions of reagents/Abs and all washes were carried out in phosphate-buffered saline (PBS). Cells on slides were fixed for 15 min in 4% paraformaldehyde. Fixed cells were washed three times for 5 min before being permeabilized with 0.1% Triton X-100 for 10 min. Permeabilized cells were then washed three times for 5 min and, subsequently, blocked with 3% BSA for 45 min. The blocking solution was replaced with a rabbit polyclonal anti-human LC3B Ab (LC3B D11 XP Rabbit mAb, Cell Signaling Technology, Danvers, MA, USA) and a mouse monoclonal anti-human CD107a Ab (LAMP-1, eBioscience, San Diego, CA, USA), both diluted in 3% BSA, and left to incubate overnight at 4 °C. The day after, cells were washed 3 times for 5 min. The secondary Abs tetramethylrhodamine (TRITC)-anti rabbit and fluorescein isothiocyanate (FITC)-anti-mouse IgG (both from Sigma-Aldrich, St. Louis, MO, USA) were incubated for 45 min. Slides were then washed three times, and the second wash was performed with the addition of Hoechst 33258 dye (Molecular Probes, Inc., Eugene, OR, USA) for nuclear staining. Fluorescence was analyzed using a fluorescence microscope (BX52 System Microscope, Olympus, Hamburg, Germany). Image acquisition and processing were conducted using IAS 2000 software (Delta System, Rome, Italy). Analyses of cellular expression were carried out at 50X and 100X magnification. The intensity of the LC3B/LAMP-1 signal in positive cells was quantified using ImageJ software (1.48v; NIH, Bethesda, MD, USA).

### 2.5. Enzyme-Linked Immunosorbent Assay (ELISA)

At baseline, and after 2, 4, and 12 weeks of treatment with BLM, sera and lysates from PBMCs of SLE patients were collected. A commercially available ELISA kit was used to determine the concentrations of IL-18 (Human IL-18 ELISA Kit; Lifespan Biosciences, Seattle, MA, USA), according to the manufacturer’s instructions.

The intra-assay coefficient of variation (CoV) was <10%, while the inter-assay CoV was <12%. The limit of detection (sensitivity of the assay) was less than 5.6  pg/mL. Data were run in triplicate in three different assays and reported as the mean  ± SD.

### 2.6. Flow Cytometry

Flow cytometry was performed for surface phenotyping of freshly isolated PBMCs before administration of BLM, using conjugated monoclonal antibodies against human CD4, CD8, and CD14 (all from BioLegend, San Diego, CA, USA). After pre-incubation with Fc Receptor (FcR)-blocking reagent (Miltenyi Biotec, Bergisch Gladbach, Germany), PBMCs were labeled with anti-BAFF-R FITC-, anti-BCMA phycoerythrin (PE)-, and anti-TACI allophycocyanin (APC)-conjugated mAbs (all from BioLegend, San Diego, CA, USA) for 30 min on ice, in order to detect BLyS receptors on cells. As a positive control, B lymphocytes were labeled with anti-CD19 peridinin-chlorophyll-protein (PerCP)-conjugated (BioLegend, San Diego, CA, USA) and anti-BLyS receptors mAbs, as previously described, and appropriate isotype controls were used. Acquisition was performed on a FACSCalibur (BD Immunocytometry Systems, San José, CA, USA), and included 30,000–50,000 events.

For the analysis of the autophagy pathway, the CYTO-ID Autophagy Detection Kit (ENZO Life Sciences, Farmingdale, NY, USA) was used, following the manufacturer’s instructions. This kit measures autophagic vacuoles and monitors autophagic flux using a selective dye. Thus, flow cytometry can detect the fluorescence from cells where autophagic vacuoles accumulate; the higher the accumulation rate, the higher the autophagic level expressed in that specific sample. For this experiment, we distinguished CD14^+^ monocytes from the peripheral blood lymphocytes (PBLs, also referred to as CD14^−^ cells) using PerCP-conjugated monoclonal Abs against human CD14 (BioLegend, San Diego, CA, USA). Acquisition was performed on a FACSCalibur (BD Immunocytometry Systems, San José, CA, USA), and included approximately 20,000–70,000 events.

Data were analyzed using the CellQuest Pro software (BD Immunocytometry Systems, San José, CA, USA). The results were expressed as mean fluorescence intensity (MFI).

### 2.7. Statistical Analysis

Statistical analyses were performed using GraphPad Prism Version 6 (GraphPad Software, San Diego, CA, USA). Data were expressed as the mean ± SD or median (Interquartile range (IQR) 25th–75th percentile), and parametric or non-parametric tests were used according to the variable’s distribution. Student’s *t*-test was used to compare different populations. Linear regression analysis (r correlation coefficient) was employed to identify significant correlations. Correlation was examined using Spearman’s rank correlation coefficient. *p*-values < 0.05 were considered statistically significant.

## 3. Results

### 3.1. SLE patients’ Features

The characteristics of the patients included in this study are summarized in Table 1. Twenty-six Caucasian patients with active disease (median SLEDAI-2K: baseline = 4.5; 12 weeks follow-up = 3, * *p* = 0.04, Table 1) were enrolled. Cytopenia was observed in five SLE patients (19.2%), and no significant changes in blood cell composition were observed during the follow-up times in SLE patients. None of the patients had concomitant antiphospholipid syndrome, and no antiphospholipid Abs positivity was detected. The mean weekly prednisone dose was 58.9 + 32.5 mg at baseline, and did not significantly change at 2, 4, and 12 weeks. The concomitant therapies were stable during the observation period. BLM was intravenously administered at 10 mg/kg at baseline, and after 2, 4, and 12 weeks.

### 3.2. Belimumab Decreases Autophagy in PBMCs from SLE Patients

Autophagy was evaluated in PBMCs from 26 SLE patients at baseline (t0) and after 2, 4, and 12 weeks (t2, t4, t12) of treatment with BLM, and from 16 HDs. For western blot (WB) analysis, we considered LC3-II and p62 as autophagy markers: LC3-II is associated with autophagic vesicles, and p62 may serve in associating ubiquitinated proteins to the autophagic machinery, so as to allow their degradation in the lysosome. Finally, since we previously detected a constitutive impairment of autophagy in T lymphocytes from patients with SLE, associated with abnormal accumulation of α-syn aggregates [22], we used α-syn as an additional marker of autophagy.

**Figure 1:**
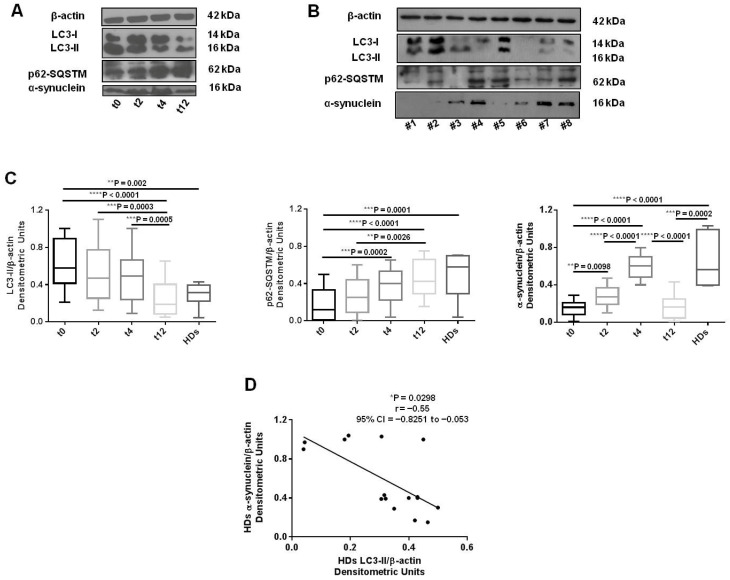
Belimumab decreases autophagy levels in PBMCs from SLE patients. (**A**) Western blot analysis of LC3-II, p62-SQSTM, and α-synuclein in lysates from SLE patients’ PBMCs at basal level (t0) and after 2, 4, and 12 weeks (t2, t4 and t12) of belimumab administration, and (**B**) from healthy donors (HDs). Results from a blot in a representative experiment are shown. (**C**) Densitometric analysis of SLE patients’ and HDs’ LC3-II (left panel), p62-SQSTM (middle panel), and α-synuclein (right panel) levels relative to β-actin, represented as the mean ± standard deviation (SD). In SLE: for LC3-II, **** *p* < 0.0001 t0 vs. t12, *** *p* = 0.0003 t2 vs. t12, *** *p* = 0.0005 t4 vs. t12; for p62-SQSTM, *** *p* = 0.0002 t0 vs. t4, **** *p* < 0.0001 t0 vs. t12, ** *p* = 0.0026 t2 vs. t12; for α-synuclein, ** *p* = 0.0098 t0 vs. t2, **** *p* < 0.0001 t0 vs. t4, t2 vs. t4, and t4 vs. t12. HDs’ mean LC3-II and p62-SQSTM expression levels are comparable to those of SLE patients at t12, and opposite (lower or higher, depending on the single marker trend) to SLE at baseline (t0) (** *p* = 0.002 for LC3-II, *** *p* = 0.0001 for p62-SQSTM, **** *p* < 0.0001 for α-synuclein). Only for α-synuclein, HDs’ expression levels are comparable to those of SLE patients at t4, given the dramatic decrease in levels observed at t12 (*** *p* = 0.0002 SLE t12 vs. HDs). (**D**) Correlation and linear regression analysis of α-synuclein and LC3-II levels relative to β-actin in HDs (* *p* = 0.0298, r= −0.55, 95% confidence interval (CI) = −0.8251 to −0.053).

We observed a gradual decrease in LC3-II levels in SLE patients PBMCs from baseline throughout the follow-up, which became significant after 12 weeks of therapy (Figure 1A,C, left panel; **** *p* < 0.0001 t0 vs. t12; *** *p* = 0.0003 t2 vs. t12; *** *p* = 0.0005 t4 vs. t12). A concomitant significant increase in p62 levels was observed (Figure 1A,C, middle panel; *** *p* = 0.0002 t0 vs. t4; **** *p* < 0.0001 t0 vs. t12; ** *p* = 0.0026 t2 vs. t12). Regarding α-syn levels, a significant increase occurred up to 4 weeks of treatment with BLM, while a dramatic decrease was observed at 12 weeks (Figure 1A,C, right panel; ** *p* = 0.0098 t0 vs. t2; **** *p* < 0.0001 t0 vs. t4, t2 vs. t4, and t4 vs. t12).

HDs’ PBMCs showed interindividual variability for the autophagy markers (Figure 1B,C) and, in particular, α-syn and LC3-II levels displayed an inverse significant correlation (Figure 1D; * *p* = 0.0298, r= −0.55, 95% confidence interval (CI) = −0.8251 to −0.053). HDs’ PBMCs displayed lower LC3-II and higher p62 expression levels in comparison with SLE patients’ PBMCs, both at baseline (LC3-II: ** *p* = 0.002 SLE t0 vs. HDs; p62: *** *p* = 0.0001 SLE t0 vs. HDs) and after BLM administration up to week 12, at which point autophagy markers’ mean levels in SLE patients were similar to those of the HDs. For α-syn, HDs’ mean expression levels were comparable to those of SLE patients after 4 weeks of therapy (t4), given that at t12, α-syn levels collapsed, returning to the basal levels (*** *p* = 0.0002 SLE t12 vs. HDs).

**Figure 2:**
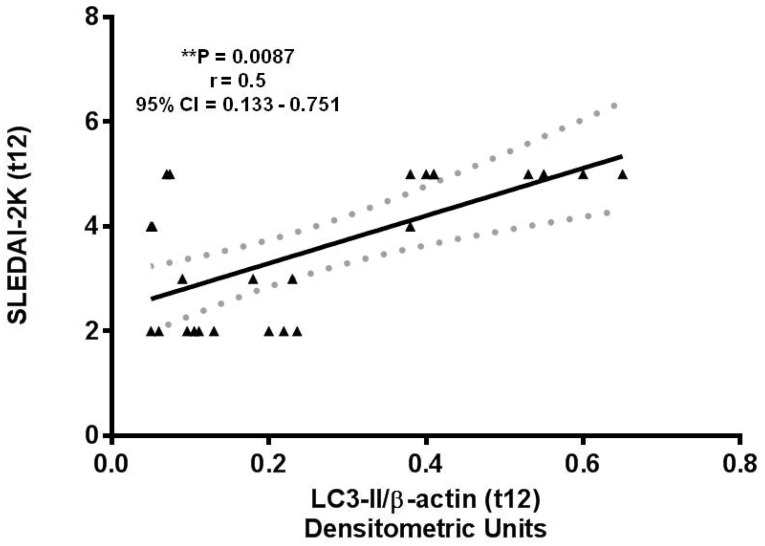
Correlation between LC3-II autophagy marker levels determined by western blotting and clinical parameters. Correlation and linear regression analysis of LC3-II expression levels in lysates from 26 patients with SLE and SLE Disease Activity Index 2000 (SLEDAI-2K) scores after 12 weeks (t12) of belimumab administration:** *p* = 0.0087, r = 0.5, 95% confidence interval (CI) = 0.133−0.751.

Interestingly, we found that LC3-II levels were significantly correlated with SLEDAI-2K at week 12 (Figure 2; ** *p* = 0.0087, r = 0.5, 95% CI = 0.133–0.751).

**Figure 3:**
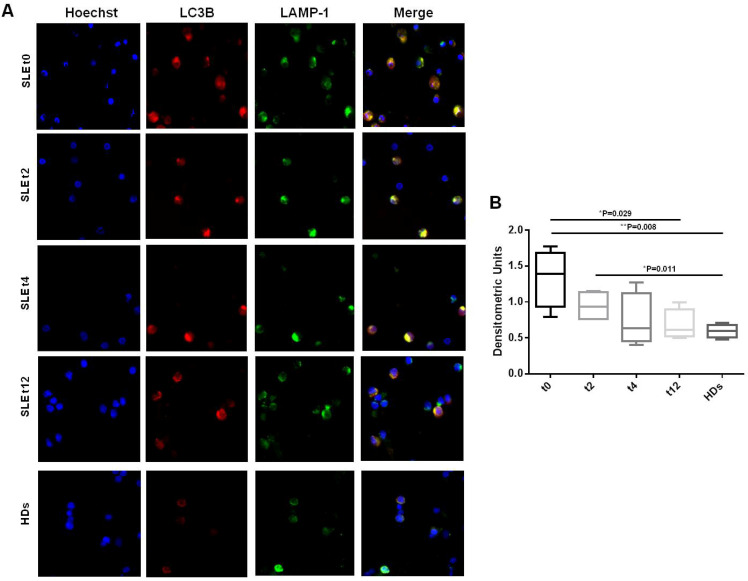
Immunofluorescence analysis of LC3B and LAMP-1 localization in PBMCs from SLE patients and HDs. A representative image of LC3B expression as LC3 puncta (red fluorescence) and LAMP-1 expression (green fluorescence) in Triton-X-100-permeated cells. PBMCs from SLE patients at (**A**) baseline (t0) and after 2, 4, and 12 weeks (t2, t4 and t12) of belimumab administration, and from HDs. Results from a representative experiment are shown. Cells were stained with Hoechst dye to reveal nuclei (blue staining). The yellow spots indicate colocalization (merge) of the two markers in autophagic cells. A diffuse cytoplasm staining with virtually no puncta is visible at t0 and t2, while at t4 and t12, a typical punctate staining is observable, more similar to that of the HDs, in which rare yellow spots (autophagic vacuoles) are present. Magnification: 50X. (**B**) Quantification by densitometric evaluation of LC3B and LAMP-1 colocalization (merge yellow spots) from positive cells, as resulting from immunofluorescent staining for each condition in 3 independent experiments. In PBMCs from SLE patients, reductions at all of the timepoints and significant differences with respect to HDs were observed (* *p* = 0.029 t0 vs. t12, ** *p* = 0.008 t0 vs. HDs; * *p* = 0.011 t2 vs. HDs). Results are represented as the mean ± standard deviation (SD) of densitometric units.

Following autophagy guidelines [23], we considered another method for monitoring autophagy. Analyzing the IF colocalization of LC3B/LAMP-1 as a hallmark of autophagy (autophagolysosomes) in PBMCs from SLE patients, we observed a significant reduction with respect to HDs at all of the timepoints, as shown in Figure 3A,B (* *p* = 0.029 t0 vs. t12; ** *p* = 0.008 t0 vs. HDs; * *p* = 0.011 t2 vs. HDs) and in Figure S1A,B (* *p* = 0.049 t0 vs. t12; * *p* = 0.021 t0 vs. HDs; * *p* = 0.012 t2 vs. t12; * *p* = 0.029 t2 vs. HDs). It should be noted that in patients’ PBMCs, LC3B/LAMP-1 colocalization (Figure 3A, merge) showed a diffuse cytoplasm staining with virtually no puncta, up to 4 (more clear in Figure S1A) and 12 weeks of treatment (Figure 3A, Figure S1A), in which a typical punctate staining more similar to that of the HDs (Figure 3A, Figure S1A) could be observed. The HDs showed very few autophagic cells, with a limited number of autophagic vacuoles (Figure 3A,B, Figure S1A,B).

Finally, in order to better characterize the autophagy process, and to preliminarily investigate in which cell population(s) the autophagic process could be affected, we performed a flow cytometric analysis, considering the autophagic vacuoles’ accumulation rate in CD14^−^ and CD14^+^ cells—PBLs and monocytes, respectively—and in total PBMCs of SLE patients. As shown in Figure S2A,B and in Table S1, we observed a gradual reduction in the accumulation of autophagic vacuoles from baseline to week 12 in all three cell populations. Furthermore, we confirmed the reduction in autophagy already demonstrated by WB and IF (CD14^−^ cells: * *p* = 0.081 for t2; * *p* = 0.031 for t4; * *p* = 0.014 for t12. CD14^+^ cells: * *p* = 0.018 for t2; * *p* = 0.019 for t4; * *p* = 0.035 for t12. PBMCs: * *p* = 0.048 for t2; *p* = 0.05 for t4; * *p* = 0.044 for t12. All of the *p*-values were calculated vs. baseline).

### 3.3. Belimumab Decreases Protein Citrullination in PBMCs from SLE Patients

**Figure 4:**
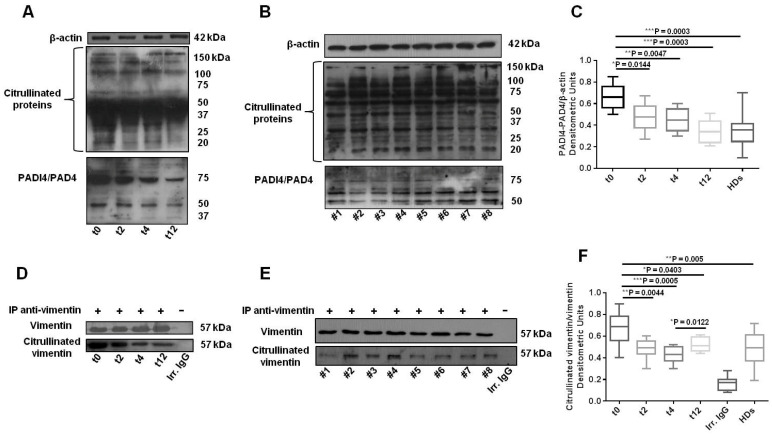
Belimumab decreases protein citrullination in PBMCs from SLE patients. (**A**) Western blot analysis of citrullinated proteins and PADI4/PAD4 enzyme levels in lysates from SLE patients’ PBMCs at baseline (t0) and after 2, 4, and 12 weeks (t2, t4 and t12) of belimumab administration, and (**B**) from healthy donors (HDs). Results from a blot in a representative experiment are shown. (**C**) Densitometric analysis of SLE patients’ and HDs’ PADI4/PAD4 expression levels relative to β-actin, represented as the mean ± standard deviation (SD). In SLE: * *p* = 0.0144 t0 vs. t2, ** *p* = 0.0047 t0 vs. t4, *** *p* = 0.0003 t0 vs. t12. HDs’ PADI4/PAD4 mean expression levels are comparable to those of SLE patients at t12, and lower than those of SLE patients at baseline (*** *p* = 0.0003 SLE t0 vs. HDs). (**D**) Western blot analysis of citrullinated vimentin immunoprecipitated from SLE patients’ PBMCs lysates at t0, t2, t4, and t12, and (**E**) from HDs. An appropriate IgG isotypic control (irrelevant IgG; Irr. IgG) was employed to verify that immunoprecipitations were correctly performed. Results from a blot in a representative experiment are shown. (**F**) Densitometric analysis of SLE patients’ and HDs’ citrullinated vimentin expression levels, represented as the mean ± standard deviation (SD). In SLE: ** *p* = 0.0044 t0 vs. t2, *** *p* = 0.0005 t0 vs. t4, * *p* = 0.0403 t0 vs. t12, * *p* = 0.0122 t4 vs. t12). HDs’ citrullinated vimentin mean expression levels are comparable to those of SLE patients at t12, and lower than those of SLE patients at baseline (** *p* = 0.005 SLE t0 vs. HDs).

PBMC lysates from SLE patients were probed with F95 Ab, which allowed us to observe the citrullinated protein bands between 43 and 65 kDa. The changes in citrullinated protein levels were first monitored by the detection of these bands. Since we observed a decreasing trend in citrullinated proteins after treatment with BLM (Figure 4A), lysates were further tested for the presence of citrullinated vimentin via immunoprecipitation—using an anti-vimentin Ab—and by subsequent immunoblotting with F95 Ab (Figure 4D). As controls, immunoprecipitates were checked for vimentin content (Figure 4D). The densitometric analysis showed a significant decrease in citrullinated vimentin levels in PBMCs from SLE patients treated with BLM (Figure 4F) after 2, 4, and 12 weeks, compared to baseline, with a trend towards increase at week 12, but remaining lower compared to baseline levels (Figure 4D,F; ** *p* = 0.0044 t0 vs. t2; *** *p* = 0.0005 t0 vs. t4; * *p* = 0.0403 t0 vs. t12; * *p* = 0.0122 t4 vs. t12).

To fully evaluate the protein citrullination process, we also assessed the 74 kDa PAD4 enzyme expression levels in PBMCs’ lysates from SLE patients treated with BLM. As shown in Figure 4A,C, we detected a significant gradual decrease in PAD4 expression at all timepoints (* *p* = 0.0144 t0 vs. t2; ** *p* = 0.0047 t0 vs. t4; *** *p* = 0.0003 t0 vs. t12).

We also tested citrullination pathway in lysates from 16 HDs’ PBMCs. As for autophagy, we found an interindividual variability for PAD4 enzyme and citrullinated vimentin levels (Figure 4B,C,E,F), expressing lower levels of markers in comparison with SLE patients’ PBMCs at baseline (t0) and after 2 and 4 weeks of BLM therapy, but similar to the SLE patients’ PBMCs after 12 weeks of treatment. Similarly to autophagy, with respect to SLE patients at baseline, HDs showed significantly lower PAD4 and citrullinated vimentin levels (*** *p* = 0.0003 and ** *p* = 0.005, respectively).

### 3.4. Belimumab Decreases IL-18 Concentration in Sera from SLE Patients

**Figure 5:**
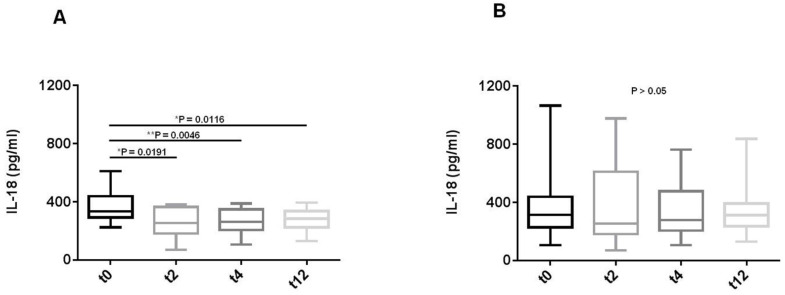
Belimumab reduces circulating IL-18 concentrations evaluated in sera from SLE patients. IL-18 levels in sera and lysates from 26 SLE patients were assessed using a commercially available ELISA kit. Sera and lysates were collected at baseline (t0) and after 2, 4, and 12 weeks (t2, t4, and t12) of belimumab therapy. All of the samples were run in triplicate in three different assays, and the results are shown as the mean ± standard deviation (SD). (**A**) For systemic IL-18 concentrations in sera, * *p* = 0.0191 t0 vs. t2; ** *p* = 0.0046 t0 vs. t4; * *p* = 0.0116 t0 vs. t12. (**B**) For IL-18 expression levels analyzed in lysates, *p* > 0.05 for all the conditions.

IL-18 is a pro-inflammatory cytokine mainly produced by monocytes, macrophages, osteoblasts, keratinocytes, and epithelial cells [24]. Because the autophagy mechanism seems to play a role in determining the release of IL-1 family cytokines such as IL-18 [25], and IL-18 was recently considered to play a role in some clinical manifestations of SLE [20], we evaluated the circulating levels of this cytokine in sera and the expression levels in PBMCs’ lysates from patients with SLE before and after 2, 4, and 12 weeks of treatment with BLM. Immediately after 2 weeks of treatment with BLM, we observed a significant reduction in IL-18 serum concentration, which remained stable at the subsequent timepoints (Figure 5A; * *p* = 0.0191 t0 vs. t2; ** *p* = 0.0046 t0 vs. t4; * *p* = 0.0116 t0 vs. t12). Conversely, lysates from PBMCs did not reveal statistical significant changes in the expression levels of IL-18 (Figure 5B; *p* = 0.9 t0 vs. t2; *p* = 0.82 t0 vs. t4; *p* = 0.84 t0 vs. t12; *p* = 0.86 t2 vs. t4; *p* = 0.79 t2 vs. t12; *p* = 0.98 t4 vs. t12).

### 3.5. Expression of BAFF-R, BCMA, and TACI on the Surface of CD4^+^, CD8^+^, and CD14^+^ Cells

**Figure 6:**
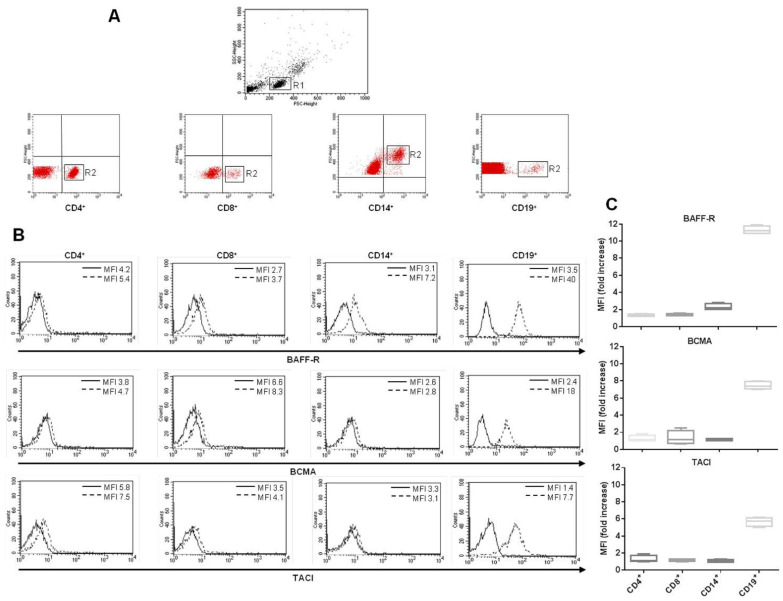
Surface expression of BAFF-R, BCMA, and TACI (BLyS receptors) on CD4^+^, CD8^+^ T cells and CD14^+^ monocytic cells of SLE patients. (**A**) Phenotypic characterization of PBMCs from 26 SLE patients at baseline (before treatment with belimumab), showing the expression of CD4, CD8, CD14, and CD19. Cytofluorimetric images show PBMC populations (R1 gate, upper panel) and CD4^+^, CD8^+^, CD14^+^, and CD19^+^ cells (R2 gate, lower panels). Results from a representative experiment are shown. (**B**) Flow cytometric analysis after staining of PBMCs with anti-BAFF-R, anti-BCMA, and anti-TACI mAbs. CD19^+^ B cells were used as positive controls for the surface expression of the receptors. Isotypic control staining is represented by the solid black line, while anti-BLyS receptors are represented by the broken black line. Results obtained in a representative experiment are shown. (**C**) Box and whisker plots indicate the levels of BLyS receptors observed in CD4^+^, CD8^+^, and CD14^+^ cells, expressed as mean fluorescence intensity (MFI). CD19^+^ cells are displayed as positive controls for the expression of BLyS receptors. Results are represented as the mean ± standard deviation (SD) of the fold increase.

The analysis of BLyS receptors’ expression on the surface of T-cell subsets and peripheral blood monocytes from SLE patients at baseline revealed the presence of BAFF-R, BCMA, and TACI at a rather low rate on CD4^+^ and CD8^+^ T cells, while CD14^+^ cells firmly expressed only BAFF-R (Figure 6A–C). As a positive control, in order to confirm the proper functioning of the Abs used for the analyses, we tested and confirmed the presence of all three BLyS receptors on CD19^+^ B cells (Figure 6A–C) from SLE patients.

## 4. Discussion

The present study demonstrated for the first time the possible impact of BLM on both autophagy and citrullination ex vivo in PBMCs from SLE patients.

B lymphocyte stimulator (BLyS)—a soluble ligand of the tumor necrosis factor (TNF) cytokine family—is a key factor for B-cell differentiation, homeostasis, and selection, and can affect cell survival signals. BLyS is the sole ligand for BAFF-R, whereas TACI and BCMA can bind either BLyS or another TNF family ligand, known as A proliferation-inducing ligand (APRIL). These receptors are mainly expressed in B cells, but different studies have also described their presence in dendritic, endothelial, and T cells’ surface [26,27]. By specifically antagonizing the modulatory protein, BLyS-targeted agents provide an effective way to control B-cell activity [16]. BLM (anti-BLyS) is a fully human monoclonal IgG1 Ab (the first approved for the treatment of SLE) that binds soluble BLyS and inhibits its binding to TACI, BCMA, and BAFF-R [16].

Recently, Spinelli et al. [26] showed that BLM was able to inhibit the apoptotic effect induced by BLyS in in vitro studies on endothelial progenitor cells from SLE patients, highlighting the possible role of BLM in determining the fate of cells other than B cells. Consequently, we investigated possible changes in the autophagy cell survival process, and in citrullination—an autophagy-related PTM, in PBMCs from SLE patients.

We observed a decrease in SLE PBMCs’ autophagy, as demonstrated by the significant reduction in LC3-II levels detected by WB after 12 weeks of treatment (t12). IF analysis confirmed these data; in particular, patients’ PBMCs showed a diffuse LC3B/LAMP-1 cytoplasm staining (colocalization), with virtually no puncta, up to t12, when a more discrete distribution of the two signals (typical puncta staining), approximately similar to that of the HDs, was observed. This aspect seems to be shared with other pathological conditions, such as some human cancers [28]. SLE PBMCs tended to stain more intensely than normal PBMCs, and HDs’ PBMCs showed very few autophagic cells, in which rare autophagic vacuoles were visible.

We also confirmed the autophagy reduction observed in SLE PBMCs via flow cytometric analysis. In addition, we performed a preliminary analysis investigating the contribution of distinct cells’ populations. Both PBLs and monocytes significantly contributed to the reduction in autophagy, even if CD14^+^ monocytes would seem to have been the main cell population in which autophagy took place. Additional studies are needed for evaluating the contribution of single cells’ subsets to the autophagy pathway.

Rockel et al. [18] reported that, when inhibiting BAFF from binding to its receptors, downstream signaling via IKK, JNK1, and NF-κB was reduced, thus decreasing autophagy. As a result, in competing with BAFF/BLyS for binding to its receptor, BLM may interfere in the autophagy regulation pathway [18]. In WB analysis, concurrent with the decrease in LC3-II, we observed an increase in p62 levels during the observation time. Since p62 binds directly to LC3 via a specific sequence motif, and is degraded during the elongation phase of autophagy [29], it can be used as a marker to study autophagic flux [23,30]. Thus, the decrease in LC3-II levels can be associated with p62 accumulation. In addition, α-syn levels also increased up to 4 weeks of BLM therapy. In a previous study, we reported that the percentage of autophagic cells was significantly higher in *α-syn*-knockdown T cells [22], suggesting that α-syn may play a direct inhibitory role in SLE T-cell autophagy. Results from another study also highlighted that T lymphocytes from SLE patients were not able to establish a response under stimulation of autophagy, and that this could be attributable to an autophagy defect [22,31]. In general, as also reported in other studies [28], relatively little is known about autophagy levels under healthy conditions; thus, establishing a reference value is challenging. As controls, we also analyzed LC3-II and α-syn levels in PBMCs of HDs, confirming the interindividual variability of these markers’ expression levels, along with an inverse correlation, as already observed in our previous study analyzing T lymphocytes [22,31]. In the present study, PBMCs from HDs’ generally displayed lower expression levels of autophagy markers in comparison with SLE patients, but exhibiting mean levels similar to the week 12 SLE PBMCs’ values. The decrease in autophagy after BLM treatment may be a sign that this drug could “restore” a “response ability” towards stimuli in SLE patients’ PBMCs. The correlation between LC3-II levels and SLEDAI-2K after 12 weeks of treatment can lead to the hypothesis of a contribution to autophagic mechanism.

In particular, we noted that the α-syn expression levels in SLE patients’ PBMCs went back to approximately their basal levels at week 12. At the cellular level, α-syn is present in synaptic terminals, mitochondria, the endoplasmic reticulum, and the endolysosomal system [32,33]. Thus, we also expected a physiological basal α-syn expression in the PBMCs of patients before the therapy with BLM. In addition, SLE patients display a defective autophagy, associated with abnormal accumulation of α-syn aggregates, as we previously demonstrated [22]. Studies at a neuronal level showed that α-syn is degraded not only by autophagy, but also by the proteasome [34]. From this point of view, α-syn levels could generally decrease faster than those of the other markers. This event could also occur in PBMCs from SLE patients. At the same time, given the half-life of BLM (~20 days), we could hypothesize a decrease in the drug’s effects, somehow competing in the influence on α-syn levels.

It should be noted that hydroxychloroquine is an inhibitor of autophagy (impairing the autophagosome–lysosome fusion and the degradative activity of the lysosome) [35], used in the management of several autoimmune diseases, such as SLE and RA. In this study, all patients were taking hydroxychloroquine at a stable dose throughout the follow-up. Therefore, we can consider this to be a uniformly distributed variable in the patient population, and we can exclude its influence in the assessment of autophagy levels. The role of immunomodulatory drugs in autophagy and citrullination is controversial. The autophagy pathway seems to be regulated in different ways, depending on the cell type and the surrounding cellular environment. On the other hand, no previous studies have reported data on the effects of drug therapies on the citrullination pathway. In the present study, all of the drugs remained stable during the 12 weeks of follow-up, excluding any effect of the treatment.

Hu et al. [27] showed that the signaling involved in BAFF-induced vitality and activation of T lymphocytes is also BAFF-R-dependent. Hence, we investigated the presence of BAFF receptors in CD4^+^ and CD8^+^ T cells and CD14^+^ monocytes. As already described in the literature, we confirmed that all cells’ subsets displayed BAFF-R on their surface; we observed a very low expression rate on CD4^+^ and CD8^+^ T cells. On the other hand, expression of TACI and BCMA has been less investigated to date [36,37]. Both TACI and BCMA were weakly expressed in CD4^+^ and CD8^+^ cells, but not in CD14^+^ cells. In line with the interplay between autophagy and immune responses and the effects of drugs targeting autophagy used in rheumatological conditions, we could speculate that the modulation of autophagy might also involve all three of these receptors in PBMCs [18]. Despite the low levels of receptors observed in PBLs, their BLyS binding capacity should not be affected, and the same applies to their functionality. Regardless, in the present study, we did not provide experimental evidence to confirm a direct impact of the receptors’ engagement on autophagy, so the hypothesis that other molecular mechanisms could also interfere with and mediate the observed effects is plausible.

To date, there are few studies investigating citrullination in SLE. Ireland and Unanue [38] suggested that citrullination should be considered to be a marker of autophagy, since citrullination in the autophagosome may promote catabolism of the proteins. Furthermore, they observed a T-cell response restricted to citrullinated peptides [15], demonstrating that citrullination can generate neoepitopes, evoking an immune response that may be involved in autoimmunity. Up to now, no evidence has been reported concerning a possible impact on citrullination after the binding of BLM to BLyS receptors, and our findings also do not suggest this. The aim of this study was the understanding of a possible connection between autophagy and citrullination in SLE patients being treated with BLM. Given the well-documented close link between the two mechanisms, changes in the levels of protein citrullination could be interpreted as being combined with changes in autophagy levels. 

Moreover, citrullination is commonly considered to be a hallmark of inflammation. The chronic inflammation and the production of autoantibodies in SLE—such as ACPA—result in a wide range of clinical manifestations, including arthritis, skin rashes, renal failure, and central nervous system damage. Moreover, PAD4 seems to be widely implicated in the pathogenesis of inflammation [39] and, in turn, inflammation can enhance the expression levels of this enzyme, so that the pathogenetic effects can take place [40]. In view of these aspects, the investigation of the protein citrullination pathway can assume a suggestive and crucial role. By monitoring citrullinated proteins—and then the citrullinated-vimentin-specific levels—in lysates from PBMCs of SLE patients, we observed a significant decrease in protein citrullination after BLM therapy. Vimentin is known to be a “target” of citrullination in inflammatory/disease conditions, and can act as a modulator in inflammatory responses [41]. After BLM treatment, a significant reduction in citrullinated vimentin immunoprecipitated from patients’ PBMC lysates was observed. Concurrently, we observed a decrease in PAD4 levels, consistent not only with the trend in citrullination levels, but also likely with a reduction in inflammation, as further demonstrated by the reduction in IL-18 concentrations in patients’ sera. As with autophagy, in HDs’ PBMCs we found an interindividual variability for citrullinated vimentin and PAD4 enzyme expression levels, observing lower mean expression levels in comparison with SLE patients’ PBMCs at baseline and after 2 and 4 weeks of BLM treatment, but comparable to the SLE patients’ PBMCs after 12 weeks.

BLM seemed to be able to decrease circulating IL-18 concentrations without affecting IL-18 expression levels evaluated in lysates from SLE patients. IL-18 serum levels already showed a fast and significant reduction after 2 weeks of BLM treatment, and remained stable throughout the follow-up. Conversely, LC3-II and p62 WB levels underwent a significant change afterwards, even if we can observe their gradual change starting from 2 weeks. In this regard, several studies showed higher IL-18 serum levels in SLE patients [20], and reported autophagy as being a possible pivotal actor in modulating IL-1β and IL-18 release [25]. It is reasonable that the reduction in circulating IL-18 could reflect the general therapeutic effects of BLM on the inflammatory condition, and these effects could also be mediated by the autophagy/citrullination reduction. On the other hand, the lack of correlation between autophagy markers and systemic IL-18 concentrations, together with the unchanged IL-18 expression levels in SLE patients’ PBMCs, could imply that other biological processes might also influence IL-18 production.

## 5. Conclusions

This study, for the first time, provides experimental evidence linking BLM, the machinery of autophagy, and protein citrullination in PBMCs from SLE patients. In particular, BLM seems to be able to control the inflammation associated with SLE, resulting in a decrease in PBLs’, monocytes’, and total PBMCs’ autophagy and citrullination levels, and in lower IL-18 serum concentrations. Considering the significant correlation between autophagy and SLEDAI-2K after 12 weeks of therapy, the results of this study suggest that BLM could further improve SLE disease activity, exerting a role on cell survival mechanisms, while simultaneously offering an opportunity for a deeper understanding of autophagy and citrullination in this disease, and providing a possible tool for the clinical management of SLE. Given the results obtained in this study, the analysis of these survival mechanisms in PBMCs could be considered to be the “harbinger” for the evaluation of single-cell subsets’ contributions to autophagy and citrullination in subsequent studies.

## Figures and Tables

**Table 1:** Demographic, clinical, and therapeutic features of SLE patients (*n* = 26).

Characteristics	Value
**Demographic parameters**	
Sex, female/male	24/2
Age, median (25th–75th percentile), years	46 (39–51)
Disease duration, median (25th–75th percentile), months	270 (105–372)
**Indication for belimumab therapy**	
Musculoskeletal involvement, *n* (%)	20 (76.9%)
Cutaneous involvement, *n* (%)	5 (19.2%)
Renal involvement, *n* (%)	1 (3.9%)
Baseline SLEDAI-2K, median (25th–75th percentile)	4.5 (4–6.5)
12-week follow-up SLEDAI-2K, median (25th–75th percentile)	3 (2–5) *
**Concomitant treatments**	
Hydroxychloroquine, *n* (%)	24 (92.3)
Mycophenolate, *n* (%)	7 (26.9)
Azathioprine, *n* (%)	6 (23.1)
Cyclosporine, *n* (%)	4 (15.4)
Methotrexate, *n* (%)	3 (11.5)
Thalidomide, *n* (%)	1 (3.9)
Glucocorticoids, *n* (%)	26 (100%)

* *p* = 0.04; SLEDAI-2K: Systemic Lupus Erythematosus Disease Activity Index 2000.

## Data Availability

All data are contained within the article and Supplementary Materials. They are available on request from the corresponding author (T.C.), and can be accessed with a valid reason.

## Supplementary Materials

The following supporting information can be downloaded at: https://www.mdpi.com/article/10.3390/cells11020262/s1: Figure S1: Immunofluorescence analysis of LC3B and LAMP-1 localization in PBMCs from SLE patients and HDs; Figure S2: Flow cytometric analysis of autophagic vacuoles accumulation in total PBMCs, PBLs (CD14^−^ cells), and monocytes (CD14^+^ cells) from SLE patients being treated with belimumab; Table S1: Flow cytometric analysis of autophagic vacuole accumulation in total PBMCs, PBLs (CD14^−^ cells), and monocytes (CD14^+^ cells) from SLE patients being treated with belimumab.
