# Supplementary material for: Belimumab Decreases Autophagy and Citrullination in Peripheral Blood Mononuclear Cells from Patients with Systemic Lupus Erythematosus
Source: Cells. 2022 Jan 13;11(2):262. doi: 10.3390/cells11020262 (PMC8773843; doi:10.3390/cells11020262)
Supplement: Supplementary file 1 [file cells-11-00262-s001.zip › cells-1544676-supplementary.pdf]

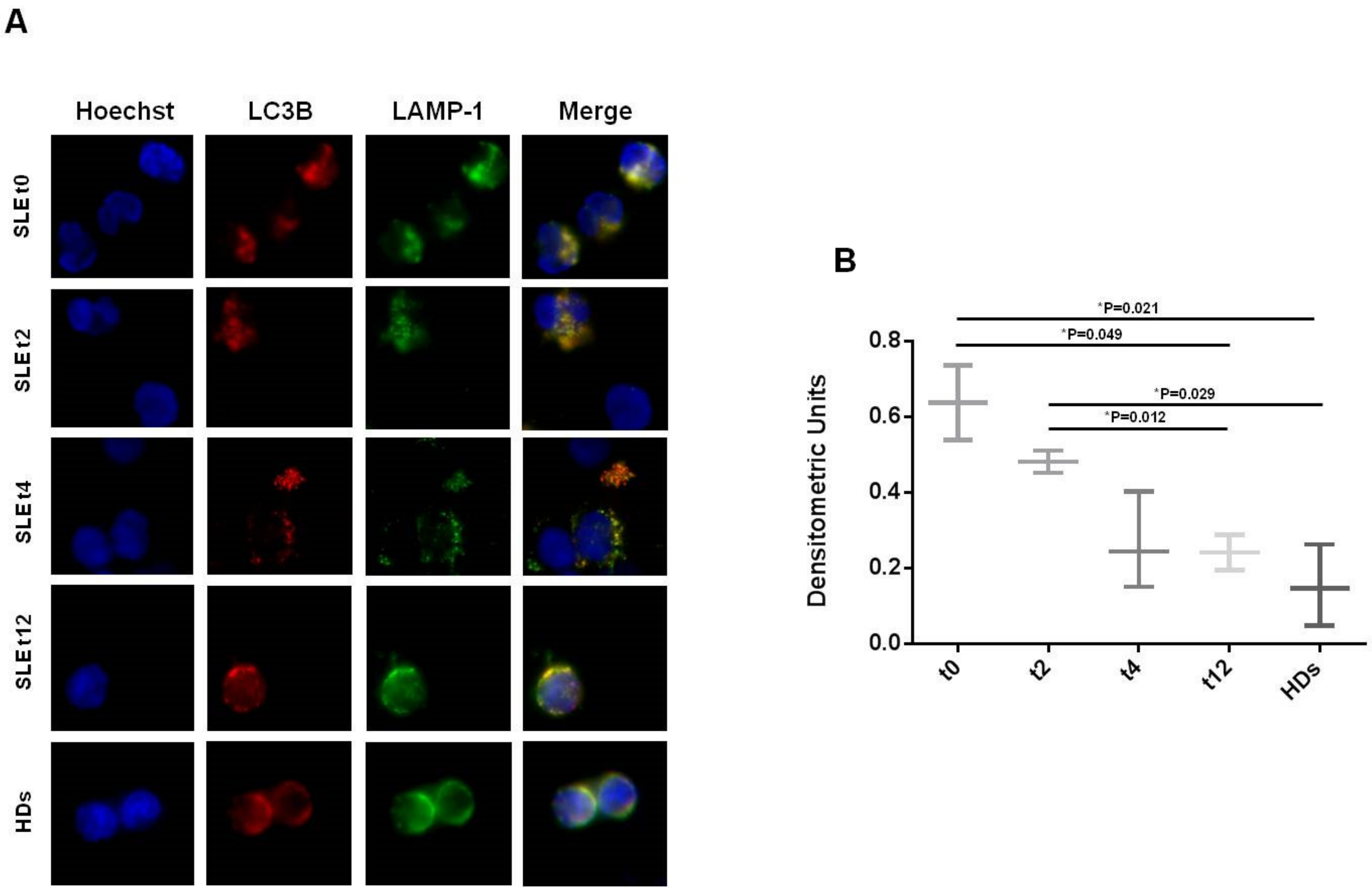


**Supplementary Figure S1.** Immunofluorescence analysis of LC3B and LAMP-1 localization in PBMCs from SLE patients and HDs. A representative image of LC3B expression as LC3 puncta (red fluorescence) and LAMP-1 expression (green fluorescence) in Triton X-100-permeated cells. PBMCs from SLE patients at (**A**) baseline (t0) and after 2, 4 and 12 weeks (t2, t4 and t12) of belimumab administration and from HDs. Results from a representative experiment are shown. Cells were stained with Hoechst dye to reveal nuclei (blue staining). The yellow spots indicate co-localization (merge) of the two markers in autophagic cells. A diffuse cytoplasm staining with virtually no puncta is visible at t0 and t2, while at t4 and then at t12, a typical punctate staining is observable, more similar to that of the HDs, in which rare yellow spots (autophagic vacuoles) are present. Magnification: 100X. (**B**) Quantification by densitometry evaluation of LC3B and LAMP-1 co-localization (merge yellow spots) from positive cells, as resulted by immunofluorescent staining for each condition in 3 independent experiments. In PBMCs from SLE patients, a reduction at all the timepoints and significant differences with respect to HDs were observed (P=0.049 t0 *vs* t12, P=0.021 t0 *vs* HDs, P=0.012 t2 *vs* t12, P=0.029 t2 *vs* HDs). Results are represented as mean ± standard deviation (SD) of densitometric units.

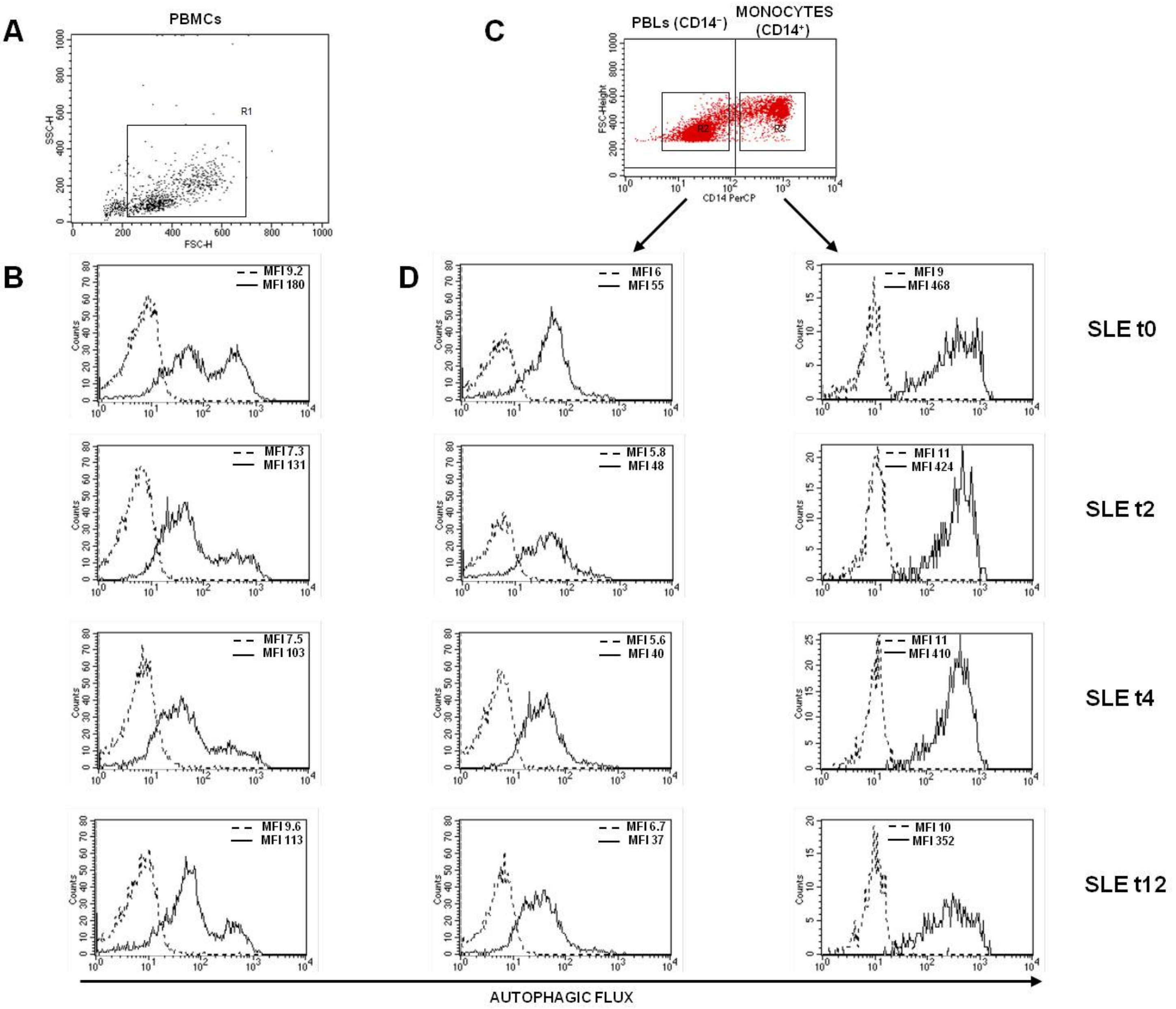


**Supplementary Figure S2.** Flow cytometric analysis of autophagic vacuoles accumulation in total PBMCs, PBLs (CD14$^-$ cells) and monocytes (CD14$^+$ cells) from SLE patients being treated with belimumab. (**A**), (**C**) Phenotypic characterization of PBMCs from 26 SLE patients by showing the expression of PBLs (CD14$^-$ cells) and monocytes (CD14$^+$ cells). Flow cytometric images represent PBMCs population (R1 gate) and PBLs (CD14$^-$ cells, R2 gate, left quadrant) and monocytes (CD14$^+$ cells, R3 gate, right quadrant). Results from a representative experiment are shown. (**B**), (**D**) Flow cytometric analysis of cells stained with a selective dye monitoring autophagic flux and anti-CD14 mAbs. Autofluorescence is represented by the black-broken line and autophagic vacuoles accumulation by the black-solid line. Results obtained in a representative experiment are shown as mean fluorescence intensity (MFI).

**Table S1.** Flow cytometric analysis of autophagic vacuoles accumulation in total PBMCs, PBLs ($CD14^{-}$ cells) and monocytes ($CD14^{+}$ cells) from SLE patients being treated with belimumab.

| | **Autophagic vacuoles accumulation (Mean Intensity Fluorescence) Fold increase** | ***P* value vs baseline (t0)** |
|---|---|---|
| **t0** | **Median (25°-75° percentile)** | |
| PBMCs | 14.1 (7.3-15.6) | |
| PBLs ($CD14^{-}$ cells) | 11.3 (7.7-15) | |
| Monocytes ($CD14^{+}$ cells) | 51 (22-51.5) | |
| **t2** | **Median (25°-75° percentile)** | |
| PBMCs | 13.7 (11.8-15.7) | ***0.048** |
| PBLs ($CD14^{-}$ cells) | 8.2 (6.7-9) | 0.081 |
| Monocytes ($CD14^{+}$ cells) | 33.5 (15.8-36.7) | ***0.018** |
| **t4** | **Median (25°-75° percentile)** | |
| PBMCs | 8.4 (8-15) | 0.05 |
| PBLs ($CD14^{-}$ cells) | 6.3 (4.5-8.8) | ***0.031** |
| Monocytes ($CD14^{+}$ cells) | 32 (27-37) | ***0.019** |
| **t12** | **Median (25°-75° percentile)** | |
| PBMCs | 8.3 (5.9-17.7) | ***0.044** |
| PBLs ($CD14^{-}$ cells) | 5.9 (5.9-6.5) | ***0.014** |
| Monocytes ($CD14^{+}$ cells) | 29.3 (25-33) | ***0.035** |
